# A Rare Case of Mediastinal Bronchogenic Cyst Infected by* Salmonella enteritidis*

**DOI:** 10.1155/2018/9121389

**Published:** 2018-05-09

**Authors:** Jasleen Kaur, Philip J. McDonald, Ravinder D. Bhanot, Reda A. Awali, Sorabh Dhar, James Rowley

**Affiliations:** ^1^Department of Internal Medicine, Wayne State University, Detroit, MI, USA; ^2^Division of Infectious Diseases, Department of Internal Medicine, Wayne State University, Detroit, MI, USA; ^3^Division of Pulmonary, Critical Care and Sleep Medicine, Department of Internal Medicine, Wayne State University, Detroit, MI, USA

## Abstract

Bronchogenic cysts are rare congenital malformations which arise from abnormal budding of the primitive tracheobronchial tube and can localize to either the mediastinum or lung parenchyma. They remain clinically silent in most adults unless they become infected or are large enough to compress adjacent structures. Infections involving bronchogenic cysts are often polymicrobial. Gram-positive, Gram-negative, and mycobacterial infections have been reported, though frequently a pathogen is not identified. We present the case of a 46-year-old female with known history of bronchogenic cyst who presented with suspected postobstructive pneumonia. She underwent cyst excision with culture positive for* Salmonella enteritidis*, an extremely rare finding on review of the literature. The patient recovered following a three-week course of antibiotics for extraintestinal salmonellosis.

## 1. Introduction

Bronchogenic cysts are uncommon congenital defects originating from sequestration of the ventral foregut, the antecedent of the tracheobronchial tree [1]. In many instances, they are an incidental radiographic finding in adults with the most common location being the mediastinum (65–90%) followed by lung parenchyma (15–20%) [2, 3]. These are mostly asymptomatic unless they enlarge and produce compressive symptoms leading to respiratory distress. Secondary infection can occur in a mediastinal bronchogenic cyst [4]. We report a case of a 46-year-old female with an expanding mediastinal bronchogenic cyst who presented with respiratory symptoms and was treated for postobstructive pneumonia. She subsequently underwent thoracotomy with complete excision of the cyst. Culture of the cyst fluid was positive for* Salmonella enteritidis*.

## 2. Case Presentation

**Figure 1:**
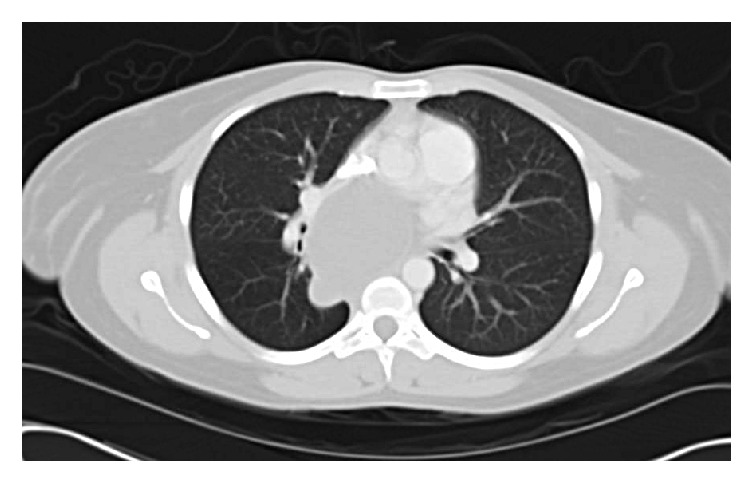
The patient's mediastinal bronchogenic cyst in 2004, measuring 4.5 × 6.5 cm.

A 46-year-old female with a history of mild persistent asthma and mediastinal bronchogenic cyst, identified incidentally in 2004 during workup of an asthma exacerbation (Figure 1), was admitted with three-day history of productive cough and fever. These symptoms were associated with dyspnea, right sided pleuritic chest pain, and cough productive of yellow sputum. Review of systems was positive for slowly progressive dysphagia for the past few months. Of note, the patient denied any recent gastrointestinal symptoms including abdominal pain and diarrhea. She denied tobacco, alcohol, and illicit drug use including marijuana.

On presentation, the patient was febrile (39.2°C), tachycardic (140 beats/min), and tachypneic (20 breaths/min) with a blood pressure of 131/88 mmHg. Physical exam revealed decreased breath sounds, rhonchi, and bronchial breathing in the right base and midlung fields. The remainder of the exam was normal. Laboratory data was remarkable for leukocytosis of 11.8 × 10^9^/L (3.5–10.6 × 10^9^/L) with a neutrophilic predominance (10.1 × 10^9^/L). Basic metabolic profile was normal and urine* Streptococcus pneumoniae *and* Legionella *urine antigens were negative. One of four blood cultures drawn in the emergency department was positive for* Micrococcus luteus *which was considered a contaminant.

**Figure 2:**
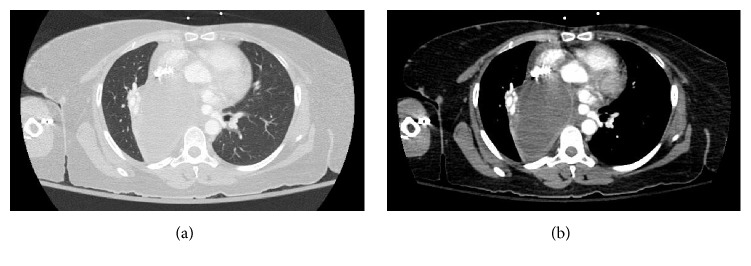
(a) The patient's large right mediastinal bronchogenic cyst displacing other mediastinal structures and compressing the right bronchus intermedius and middle/lower lobe bronchi. Lung window. (b) Mediastinal window.

Chest X-ray showed an airspace opacity in the right middle and lower lobes as well as a right hilar mass representing the previously diagnosed bronchogenic cyst. A subsequent CT scan confirmed a large right mediastinal bronchogenic cyst that had increased in size causing displacement of the mediastinal structures and compression of the right bronchus intermedius and middle/lower lobe bronchi (Figure 2). Based on these findings, the patient was diagnosed with postobstructive pneumonia and she was started on empiric ceftriaxone and azithromycin. Clindamycin was added the next day for anaerobic coverage considering that the patient had been experiencing dysphagia secondary to esophageal compression from her expanding bronchogenic cyst, and this might increase her risk of aspiration. She began to demonstrate clinical improvement within few days but continued to have fevers to 38.2°C despite normalization of her WBC count and otherwise stable vital signs. Subsequent blood cultures remained negative.

Due to the expansion of the cyst with significant compressive features, operative intervention was deemed necessary. The patient subsequently underwent an uncomplicated right sided thoracotomy, mediastinal cystic mass excision, closure of bronchus intermedius stump, and esophageal repair. Dissection of the cyst had been complicated by extensive pericystic adhesions and esophagomyotomy was required for complete excision. The patient was monitored in the surgical ICU postoperatively. A sample of the cyst contents was submitted for routine and anaerobic culture. A concurrent respiratory culture was not obtained. A non-lactose fermenting Gram-negative bacillus was isolated from the cyst fluid and identified as* Salmonella* spp. Serological identification showed that the organism was* Salmonella enteritidis*. Susceptibility testing (Table 1) revealed the isolate to be susceptible to all antibiotics tested including ceftriaxone, ciprofloxacin, and trimethoprim-sulfamethoxazole (TMP-SMX). Histological examination revealed a true bronchogenic cyst lined by ciliated columnar epithelium containing cartilage within its wall. No stool culture was obtained since the patient did not have diarrhea or other gastrointestinal symptoms during her admission.

The remainder of the patient's hospital course was uneventful. Ceftriaxone was continued while being inpatient. She was discharged on postoperative day 12 at which time she was transitioned to ciprofloxacin 500 mg PO BID to complete a three-week total antibiotic course for extraintestinal salmonellosis.

## 3. Discussion

The bronchogenic cyst is a rare congenital malformation. It results from an abnormal budding of the tracheobronchial tree when segregating from the primitive intestine, around the seventh week of gestation [1]. Depending on the time of separation from the primary airways, bronchogenic cysts may present as mediastinal cysts (early separation; majority of the cases; almost 65%) close to the tracheobronchial tree or as pulmonary cysts within the lung parenchyma (late in gestation; third trimester) [1, 5]. Tracheobronchial compression and life-threatening symptoms due to cyst enlargement are common in the pediatric population because of the relatively soft tracheobronchial tree. In contrast, most bronchogenic cysts in adults are asymptomatic and are often incidental radiographic findings. However, these patients may eventually develop potentially serious symptoms if the cysts become infected, rupture, bleed, or undergo malignant transformation [4]. As such, the clinical presentations of bronchogenic cysts are variable with most frequent symptoms being cough, fever, chest pain, and dyspnea [4]. Unusual presentations include hemoptysis, dysphagia, pneumothorax, or superior vena cava (SVC) syndrome.

Superimposed cyst infection is usually the result of communication with the tracheobronchial tree. Intraparenchymal bronchogenic cysts are more likely to have a connection with the tracheobronchial tree than mediastinal cysts and are thus more prone to infectious complications [4, 6]. Mediastinal cysts, on the other hand, rarely fistulize with the bronchi or lung parenchyma and thus seldom become infected. In one of the largest reported series of 86 patients, St-Georges et al. found only one infected mediastinal cyst; the source of infection was unknown as this cyst was not fistulized [7]. The radiological data and operative details of our patient confirmed a mediastinal cyst with no connection to the tracheobronchial tree. The fact that, despite this, it became secondarily infected makes it an extremely unusual presentation.

Microbiological data from infected cysts frequently reveals polymicrobial infection with both Gram-negative and Gram-positive bacteria. There have been three reported cases in which the purulent contents of bronchogenic cysts grew pneumococcus, other* Streptococcus* spp. and* E. coli* [4]. There have been two reports of* H. influenzae* infection of an existing cyst [8, 9]. Nontuberculous* Mycobacterium* species including* M. kansasii *and* M. avium* complex (MAC) have also been isolated from infected bronchogenic cysts and lung bullae [10, 11]. The causative pathogen or pathogens are often unidentified, and the frequency of infection by specific pathogens has not been elucidated due to the rarity of this condition. Our patient's mediastinal bronchogenic cyst became infected with* Salmonella enteritidis, *an occurrence which, to our knowledge, has only been reported once before. A single case has been described in a previously healthy 24-year-old man who developed respiratory symptoms three days after an episode of gastroenteritis. He underwent thoracotomy and excision of a mediastinal cyst which was positive for* S. enteritidis*, as was his stool [6].

Salmonellae are Gram-negative, non-lactose fermenting, facultatively anaerobic, motile bacilli. The manifestations of salmonellosis are classified into four categories: gastroenteritis, enteric fever, focal infection, and a chronic carrier state [12]. Nontyphoidal* Salmonella* causes 93 million enteric infections per year worldwide, 3.4 million of which lead to invasive disease (49 cases per 100,000 people) [13]. Approximately one percent of enteric infections with nontyphoidal* Salmonella* result in bacteremia, though the actual number may be higher as many primary infections are unrecognized or not diagnosed microbiologically. Primary* Salmonella* bacteremia in the absence of gastrointestinal symptoms may be an initial sign of undiagnosed immunodeficiency since risk factors include HIV, TNF blockade, transplantation, congenital immune defects, malignancy, diabetes, liver disease, hemoglobinopathies, and extremes of age [14]. Once it migrates from the gut to the lymphatics and finally to the bloodstream,* Salmonella *can disseminate to any site causing serious and potentially life-threatening infections including empyema, pyelonephritis, osteomyelitis, endocarditis, meningitis, or abscess [15]. In one case series of urban Thai children, the presence of pneumonia was associated with increased mortality risk [16].

In both our case and that presented by Kostopoulos et al.,* Salmonella enteritidis *most likely disseminated hematogenously to an isolated mediastinal cyst. Interestingly, no immunocompromising condition was identified in either of those patients. Our patient declined HIV testing but had no other apparent risk factor for salmonellosis such as those mentioned above. Until her cyst was determined to be mediastinal rather than intraparenchymal, another route of acquisition was entertained. In 1981, 85 cases of enteritis due to* Salmonella muenchen* were reported in several states, all of which were found to be related by plasmid analysis [17]. Inhalation of contaminated marijuana was found to be the route of acquisition rather than a food source. Our patient denied marijuana use. She likely experienced an episode of* Salmonella* enteritis at some point in the past and became a chronic carrier, as do an estimated 0.2–0.6 percent of otherwise healthy individuals after such an infection [18]. Eradication is not routinely recommended for chronic carriage outside the setting of immunocompromise. Extraintestinal salmonellosis itself generally requires surgical drainage or debridement in addition to antibiotics [12, 15]. Reasonable empiric antibiotic therapy includes a third-generation cephalosporin, a fluoroquinolone (e.g., ciprofloxacin, levofloxacin), TMP-SMX, or ampicillin. Susceptibility testing should be performed on the organism to inform definitive therapy given increasing fluoroquinolone and cephalosporin resistance. A two-week antibiotic course is appropriate for most individuals with primary bacteremia, or three weeks following drainage/debridement of a known focus of infection. Longer courses of 4–12 weeks can be considered depending on the adequacy of debridement, presence of prosthetic material, immune status of the patient, and standard duration for certain sites of infection (e.g., endocarditis, osteomyelitis).

There exist significant differences in the current literature with respect to treatment of bronchogenic cysts. While most researchers agree that symptomatic cysts should be surgically excised, there is a lack of consensus on how to manage the majority of asymptomatic patients. A conservative approach consisting of observation or minimally invasive procedures has been advocated for small cysts. However, case series indicate that most patients eventually develop symptoms or complications related to their bronchogenic cysts. St-Georges et al. reported that 43% of patients with known mediastinal cysts for 6 months to several years eventually became symptomatic [7]. In a case series by Patel et al., three patients followed for 1.5 to 10 years ultimately required resection due to the development of symptoms [19]. Similarly, our patient was asymptomatic for many years from the initial diagnosis of her bronchogenic cyst but over time developed compressive symptoms and the infectious complications described above. This case, therefore, supports the recommendation established by large series that any suspected or diagnosed bronchogenic cyst should be excised even if it is initially asymptomatic [19, 20].

In conclusion, asymptomatic patients with known bronchogenic cysts may eventually develop symptoms and serious, life-threating complications. These cysts may become infected, either by contiguous spread of bacteria from the tracheobronchial tree or by hematogenously in the case of mediastinal cysts. Nontyphoidal* Salmonella *spp. have rarely been isolated from bronchogenic cysts, even in immunocompetent individuals. In addition to appropriate antimicrobial therapy, prompt surgical excision is imperative for source control and definitive tissue diagnosis.

## Figures and Tables

**Table 1:** Antibiotic susceptibilities of the patient's *Salmonella enteritidis* isolate.

Antibiotic	MIC in *μ*g/mL	Interpretation
Ampicillin	≤2	Susceptible
Ceftazidime	≤0.5	Susceptible
Ceftriaxone	≤0.5	Susceptible
Ciprofloxacin	≤0.5	Susceptible
Imipenem	≤0.25	Susceptible
Trimethoprim/sulfamethoxazole	≤0.5/9.5	Susceptible
